# Vulnerability of *Gubernatrix cristata* to climate change, anthropogenic pressures, and hybridization threats

**DOI:** 10.1038/s41598-025-94293-7

**Published:** 2025-04-09

**Authors:** Regina Gabriela Medina, Marisol Domínguez

**Affiliations:** 1 Departamento de Ecología, Facultad de Ciencias Biológicas, Pontificia Universidad Católica de Chile, Santiago, Chile; 2 Instituto de Biodiversidad Neotropical, (CONICET), Universidad Nacional de Tucumán, Facultad de Ciencias Naturales, Ciudad Universitaria, Horco Molle, Yerba Buena, 4107 Tucumán Argentina; 3 Unit of Evolutionary Biology/Systematic Zoology, Institute for Biochemistry and Biology, University of Potsdam, Karl-Liebknecht-Str. 24-25, Haus 26, D-14476 Potsdam, Germany; 4 Laboratorio de Ecología y Comportamiento Animal, Departamento de Ecología, Genética y Evolución, Facultad de Ciencias Exactas y Naturales, Instituto IEGEBA UBA‐CONICET, Universidad de Buenos Aires, Pabellón II, Ciudad Universitaria, C1428EHA Buenos Aires, Argentina

**Keywords:** Risk map, *Diuca diuca*, Human footprint, Protected areas, Geographic range loss, Ecological niche modeling, Conservation biology, Ecological modelling, Evolutionary ecology, Ecology, Evolution

## Abstract

Estimating extinction risk is challenging due to insufficient data on current and future threats. This study develops a framework incorporating the impacts of climate change, anthropogenic pressures, and biotic interactions for assessing extinction risks using the endangered Yellow Cardinal (*Gubernatrix cristata*) as a case study. Using ecological niche modeling (ENM) with occurrences, climate, and land use data, we projected current and future distributions of *G. cristata*, identifying key constraints for its occurrence. Field validation through a citizen science initiative contributed new presence records, supporting our model’s predictions. Currently, 4.50% of cardinal’s suitable areas overlap with areas of high anthropic pressures, while 27.04% are in contact with the hybridizing species *Diuca diuca*. Future projections predict a 60% shift in the cardinal’s distribution, exacerbating its vulnerability due to greater overlap with areas of high anthropic pressures and reduced presence in protected areas. We identified key risk areas on the distribution’s periphery, vulnerable to geographic range loss and increased interaction with *D.* *diuca* due to climate change. Targeted management actions are recommended to mitigate further degradation. This study illustrates the potential of integrating citizen science, ENM, and anthropogenic and biotic pressures to develop conservation strategies, offering a versatile, universally applicable framework crucial for global biodiversity and conservation efforts.

## Introduction

The current global biodiversity crisis, characterized by rapid species loss and escalating extinction rates, underscores the urgent need for effective conservation strategies. Unlike prehistoric mass extinctions, current extinction rates are alarmingly high, leading to an unprecedented loss of species worldwide^1^. Habitat destruction, climate change impacts, and the spread of invasive species are among the primary drivers of this crisis, significantly altering ecosystems and threatening global biodiversity^2,3^. Notably, human-induced climate change has emerged as a particularly serious threat, potentially the most critical of all ^4^, affecting the geographic availability of climatically optimal environments for various species. This could lead to significant contractions or expansions in geographic ranges, and even shifts in species distributions, as they adapt to changing conditions^5^.

Bird species worldwide, particularly those in Neotropical regions, are becoming increasingly threatened. Globally, 14% of bird species (1486 of 11,147 assessed) are at risk of extinction^6^. In the neotropics, the Thraupidae family (Order: Passeriformes) is notably impacted, with 20% of its species, including the Yellow Cardinal (*Gubernatrix cristata*), currently listed as endangered by the IUCN and facing a high risk of extinction in the near future if conservation measures are not effectively implemented^6^. This endemic bird from South America historically occupied open woodlands, savannas, and shrubby steppes in Argentina, Brazil and Uruguay^7,8^. However, its current populations have now become fragmented and isolated. Previous research has shown that these remaining cardinal populations are differentiated by their genetic^9^ and song^10^ variation. The largest remaining groups are in Argentina^7,11,12^, while in Uruguay, the population is mainly concentrated in the Río Uruguay basin and has declined to fewer than 300 mature individual^13^. In southern Brazil, the situation is even more critical, with fewer than 50 individuals observed^14^.

Yellow Cardinals face anthropogenic and biotic pressures that differ across their current distribution in diverse ways. Key challenges include the targeted capture of males for the illegal pet trade, due to their striking coloration and song^8,15,16^, and habitat loss from human activities such as deforestation^17^ and land conversion, including afforestation with *Eucalyptus* spp. plantations and conversion to cattle pasture with overgrazing^18^. Regarding biotic pressures, hybridization with the Common Diuca Finch *Diuca diuca*^10,19^, particularly in areas of sympatry, further threatens species persistence^20^. This process not only can reduce reproductive success, contributing to substantial declines in population sizes^21^ but also has the potential to profoundly alter its evolutionary trajectory if long-term genetic mixing through backcrossing between hybrids and parental species occurs^22^. Reports of hybridization have been occasional^19,23,24^, but no global effort has yet been made to map it as a potential threat. This highlights the importance of accurately mapping their geographical overlap to guide effective conservation initiatives. The geographic distribution of Yellow Cardinals was mapped^7^ using the medium propinquity method, a spatial analysis technique that assesses the relative closeness between locations based on mean distances^25^. While that study focused on comparing past and present distributions primarily based on spatial relationships without integrating environmental variables, our research extends this earlier work by using advanced correlative approaches that integrate presence points with predictive environmental variables, offering a refined prediction of suitable habitats under various future climate change scenarios. Such advancements in the development of algorithms for spatial analysis, along with rapid access to extensive biodiversity data, have enhanced our ability to predict suitable habitats effectively^26^. Central to our study, ecological niche modeling has emerged as a fundamental statistical method for predicting current and potential future suitable habitats, identifying new populations, and assessing threats^27^. Therefore, these methods greatly improve our understanding of the geographic distribution and threat dynamics of endangered species.

The main aim of this study was to develop a framework for assessing species extinction risk considering anthropic, biotic and climate change threats. For this purpose, we 1) model *Gubernatrix* *cristata’s* geographic distribution using ecological niche modeling to identify unknown populations in potentially suitable areas according to climate and land use; 2) evaluate the potential impact of climate change on predicted suitable areas considering their current human footprint and protection status; 3) estimate the current and potential future geographic overlap with *D.* *diuca* as a proxy for hybridization likelihood between them; and 4) summarize the potential risk areas based on vulnerability to geographic range loss and overlap with Diuca Finches in two separate risk maps, one for 2050 and another for 2070.

## Results

### Potential geographic distributions and field validation

One hundred and sixteen candidate models were obtained, each showing statistical significance above null expectations, indicating a nonrandom association between model predictions and observed occurrences. Among these, a single model was selected based on three criteria: statistical significance, low omission rate, and optimal AICc, incorporating linear and product features with a regularization multiplier of 0.1 (Fig. S1, and Tables S1, S2, S3). The key contributors to the model, in terms of their percentage of contribution, included two precipitation variables (annual precipitation and precipitation seasonality), land cover (shrub coverage), and two temperature variables (isothermality (bio2/bio7) (× 100), and annual mean temperature), ranked in this order (Fig. S2A). The response curves (Fig. S2B-I) gave an indication of the range of optimum variable suitability, indicating a decrease in environmental suitability associated with increases in annual precipitation, precipitation seasonality, and isothermality. The optimal value for annual precipitation (the sum of monthly precipitation values throughout the year) was approximately 100 mm, for precipitation seasonality (the variance in monthly precipitation totals across the year) of approximately 10%, and for isothermality (a measure of how day-to-night temperatures oscillate relative to summer-to-winter oscillations) of 38%, with the latter suggesting greater diurnal temperature variation relative to annual changes. Conversely, environmental suitability showed positive correlations with annual mean temperature and shrub coverage, with optimal values of 23 ºC and 100%, respectively (Fig. S2B, I).

**Fig. 1:**
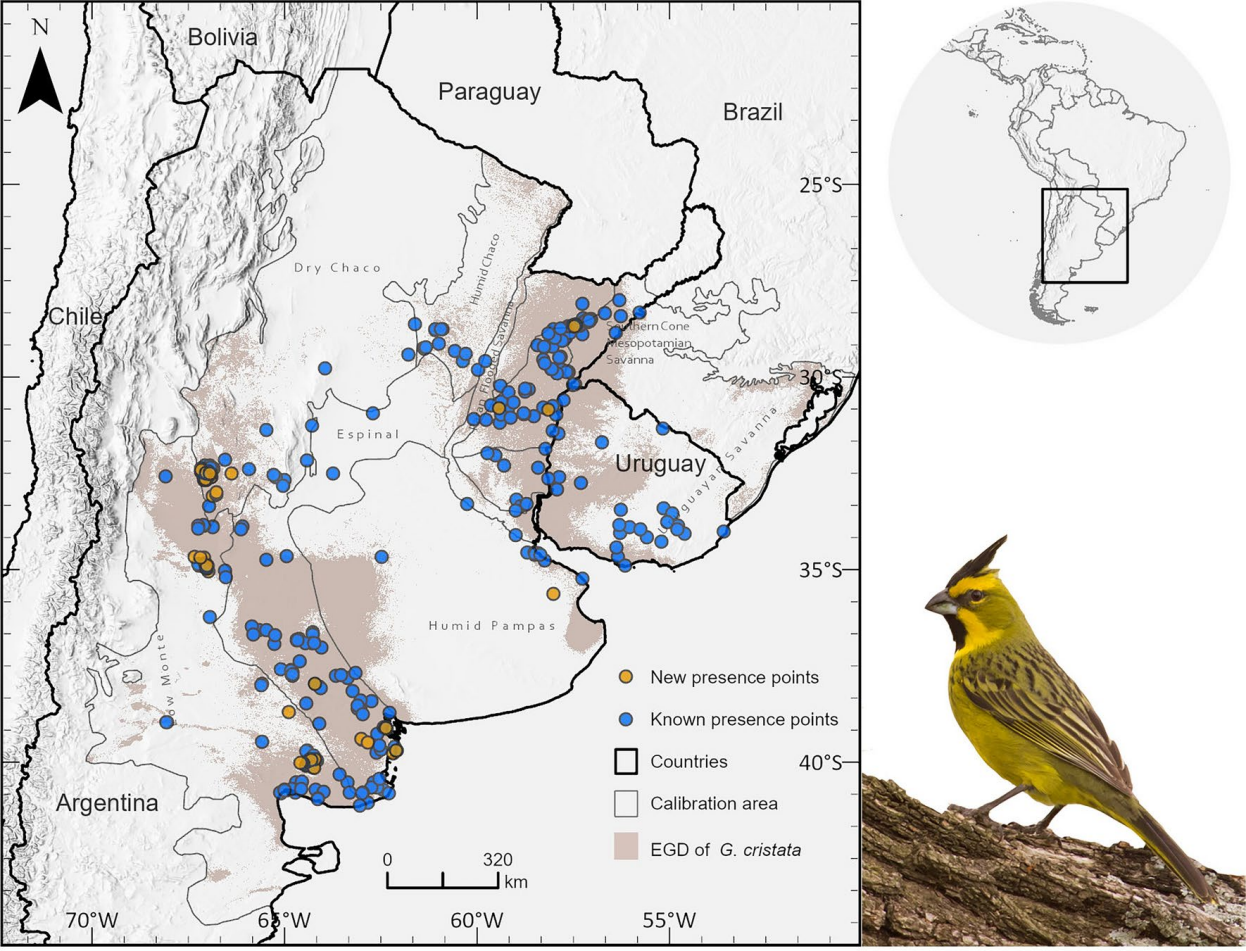
Estimated geographic distribution (EGD) of *Gubernatrix cristata* and field validation results. The EGD of *G. cristata* is indicated by the gray shaded area. Known presence points from 1950–2018, used in the ENM process, are marked with blue dots. Newly identified presence points, represented by orange dots, were gathered through a citizen science program in 2018 and were used to validate the final model. The calibration area is outlined by a gray line. Photograph of a male Yellow Cardinal, courtesy of Carlos Figuerero.

The model predicts a current geographic distribution of *Gubernatrix cristata* from latitudes –24 to –44 and longitudes –51 to –70, covering an area of approximately 855,423 km^2^, with most of the suitable environment distributed in Argentina, followed by Uruguay and then Brazil. The Yellow Cardinal distribution predominantly spans the south and north of the Espinal ecoregion, extending into the Uruguayan and Mesopotamian savanna, and reaching into the neighboring ecoregions Humid Chaco, southern Dry Chaco, and Low Monte (Fig. 1).

The citizen science program revealed 167 new Yellow Cardinal presence points within areas predicted by our model, validating our findings (Fig. 1, Table S4). While most of these new records are in proximity to the known distribution, 21 were located between 15 and 100 km from the nearest previously known record.

### Potential impact of climate change on range sizes and geographic shifts

**Fig. 2:**
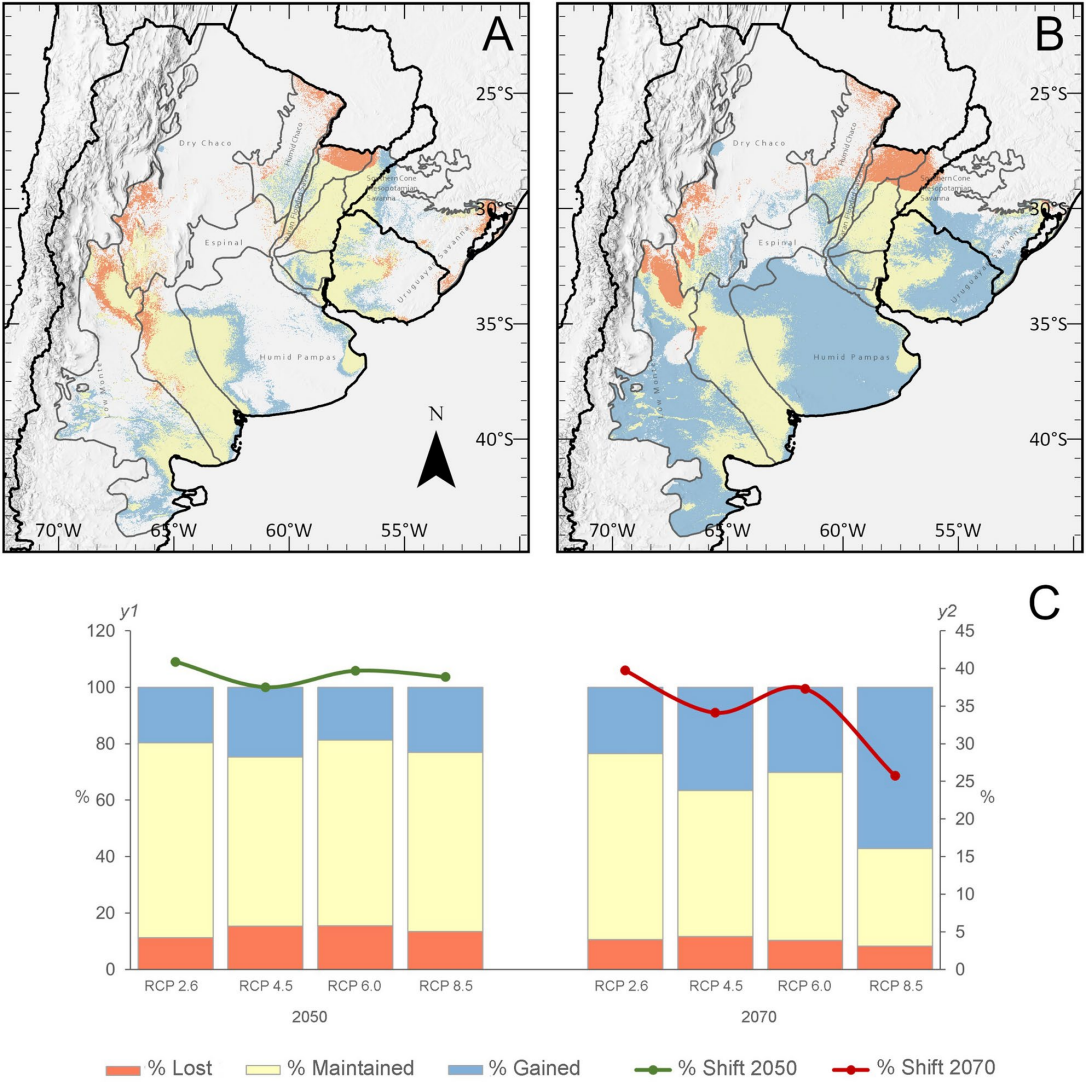
Predicted changes in *G.* *cristata*’s EGD. **A**-**B**. Projected geographic distribution of *G. cristata* under the RCP 8.5 climate scenario for 2050 and 2070, respectively. **C**. Bar plots show the percentage of environmental suitability gained (blue), lost (orange), and maintained (yellow) by *G. cristata* (*y1* exe), comparing the current scenario with each projected future scenario. Lines illustrate the overlap index (*y2* exe) between the current state and each future scenario.

Under future climate scenarios, our projections indicate changes in the estimated geographic distribution (EGD), including areas gained, lost, maintained, and geographical shifts, in varying proportions depending on the scenario and time period (Fig. S3). By 2050, the range is expected to increase by 18.67% to 36.80%, with an average expansion of 21.51% (± 1.42%), depending on the representative concentration pathways (RCP) model. By 2070, the predicted range expansion varies between 23.43% and 57.06%, reaching its maximum under the RCP 8.5 scenario (Fig. 2). Concurrently, a decreasing geographic range in some areas is also forecasted. By 2050, this reduction varies from 11.20% to 15.53% (average of 13.86 ± 2.02%), with the largest decrease occurring under the RCP 6.0 scenario. By 2070, the expected decrease varies between 8.26% and 11.66% (average of 10.20 ± 1.42%), with the greatest drop projected under the RCP 4.5 scenario. In terms of range stability, our models show that by 2050, the proportion of the range expected to be maintained varies between 60.02% and 69.14% (average of 64.70 ± 3.83). By 2070, this range is projected to vary from 34.67% to 65.93% (average of 53.00 ± 13.51%), with the greatest stability occurring under the RCP 2.6 scenario for both periods of time. Furthermore, projections for the Yellow Cardinal’s EGDs forecast a maximum geographical shift of 61% (O index = 39%) by 2050 and up to 75% (O index = 25%) by 2070 (Fig. 2C), suggesting substantial changes in the distribution patterns of *G. cristata* over time, which is clearly visible in RCP 8.5 scenario (Fig. 2A-B).

### Anthropic pressures, state of protection, and biotic threats in current and future suitable areas

**Fig. 3:**
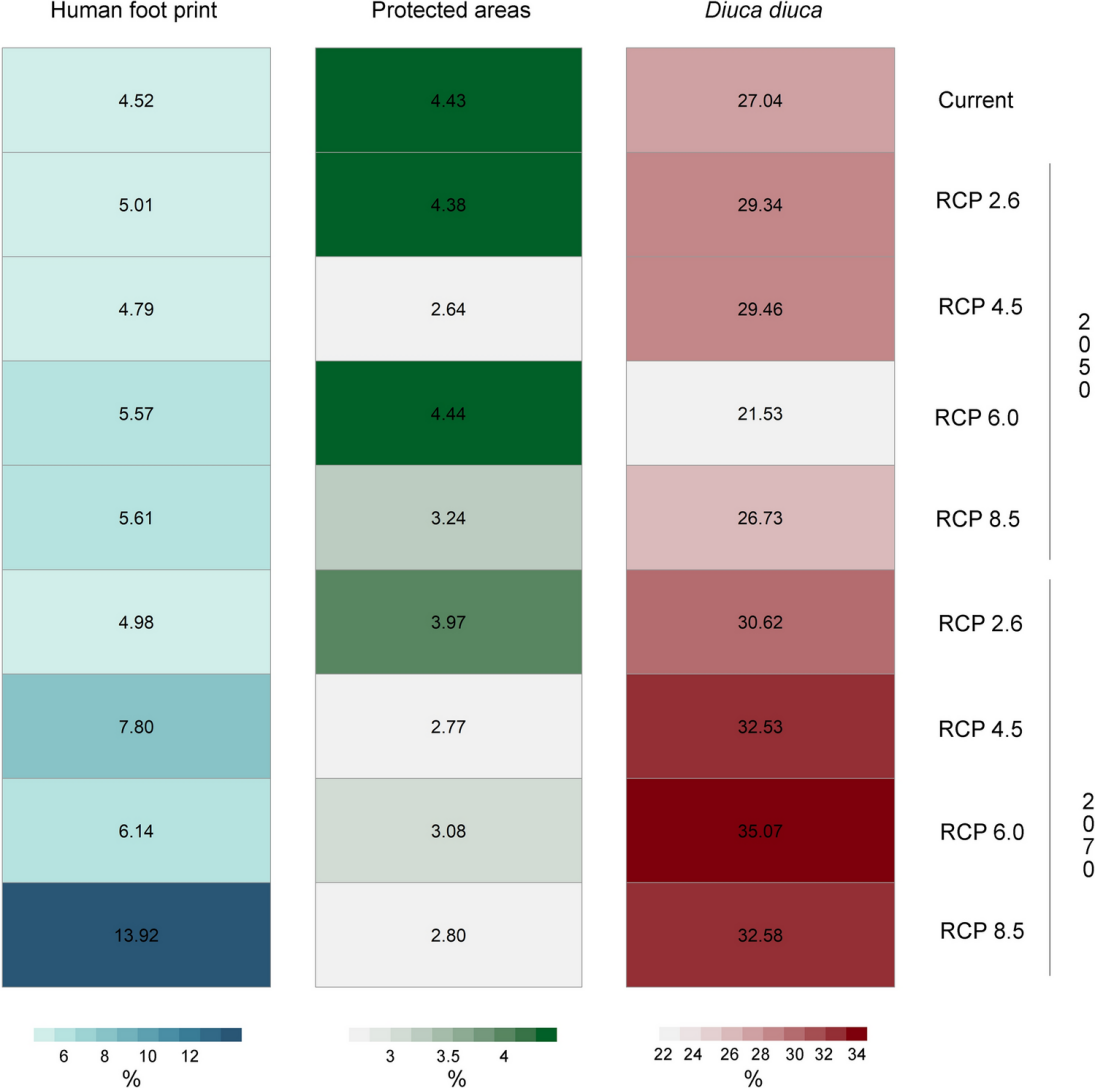
Geographic overlap of *G.* *cristata*’s current and projected EGDs with areas currently having a high human footprint index (> 10), with the current and future projected EGDs of *Diuca diuca,* and with current protected areas.

Currently, 4.5% of the Yellow Cardinal’s EGD (95,756 km^2^) is in regions with high anthropogenic pressures, primarily located in the northeast and southwest of the cardinal’s distribution, near large cities. By 2050, part of the predicted cardinal’s EGD (between 4.8% and 5.6%) is expected to lie within areas with high levels of human footprint (HFP). Notably, the greatest overlap is forecasted for 2070 under the RCP 8.5 climate change scenario (13.9%, 291,467 km^2^), followed by RCP 4.5 (7.8%, 163,621 km^2^, Fig. 3).

Our analysis revealed gaps between both the current and the predicted future distributions of Yellow Cardinals and the extent of existing protected areas (PAs). Currently, only 4.43% (23,834 km^2^) of the cardinal EGD fall within protected regions. For future scenarios, our model predicts a further decline in the overlap between *G.* *cristata’s* EGD and existing PAs, except under the RCP 6.0 scenario in 2050, where the proportion of the cardinal’s future distribution within PAs mirrors the current situation (Fig. 3). The predictions for 2050 suggest a reduction in PAs coverage ranging from 0.05% (under RCP 2.6) to 1.79% (under RCP 4.5), and for 2070, a decrease between 0.46% (under RCP 2.6) and 1.66% (under RCP 4.5, Fig. 3) is predicted.

We derived the potential geographic distribution of *Diuca diuca* from 124 candidate models, each showing a statistically significant improvement over null expectations. Of these, a single final model was selected based on three criteria: statistical significance, low omission rate, and AICc, incorporating a linear feature and a regularization multiplier of 0.1 (Table S1, S2, S3 and Fig. S4). The current predicted distribution of *D.* *diuca* spans approximately 2,854,859 km^2^ including the Espinal, Dry Chaco, High Monte, Patagonian Steppe and much of Low Monte ecoregions in Argentina, as well as the Atacama Desert and areas from the Chilean Matorral to the Valdivian Temperate Forest ecoregions in Chile. For the future climate scenarios, a general decrease in the geographic range size of *D.* *diuca* is predicted, except for the RCP 8.5 scenario in 2050. The range of diucas is predicted to decrease from 6.81% (under RCP 6.0) to 1.52% (under RCP 8.5) in 2050, and from 9.88% (under RCP 2.6) to 5.21% (under RCP 4.5) by 2070. This trend contrasts with that observed for *Gubernatrix cristata*, as we reported above.

Under current climate conditions, the geographic overlap between the EGDs of *D.* *diuca* and *G. cristata* is 27.04%. For 2050, the trend in overlap shows divergent forecasts across scenarios: it increases by 2.30% and 2.42% under RCP 2.6 and RCP 4.5, respectively, but decreases by 5.50% and 0.30% under RCP 6.0 and RCP 8.5. However, by 2070, an increase in overlap between 3.58% and 8.03% is predicted across all climate scenarios, reaching a maximum under RCP 6.0 scenario (Fig. 3).

### Potential risk maps

**Fig. 4:**
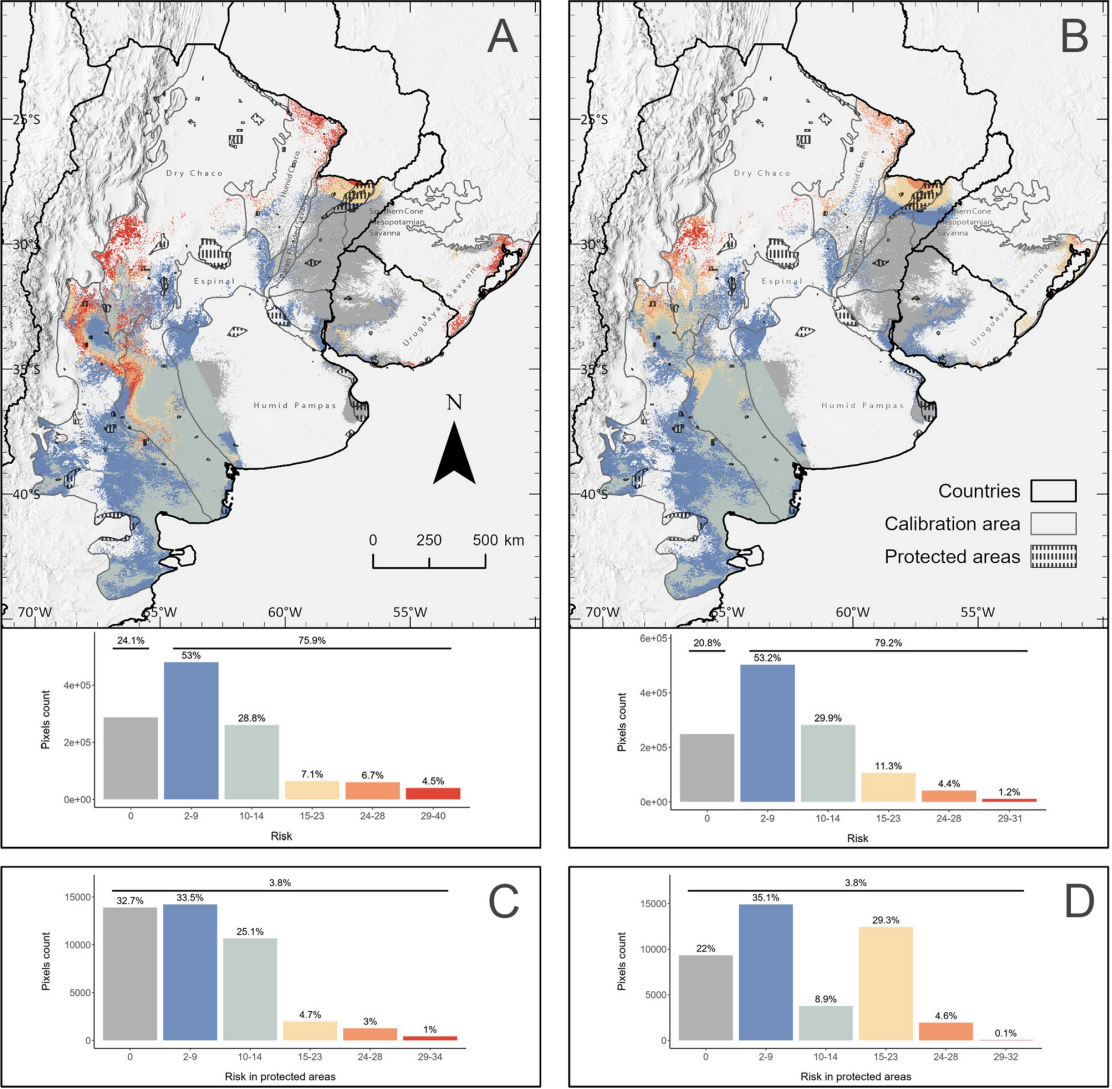
Risk maps for *Gubernatrix cristata* according to the loss of environmentally suitable areas and the contact zone with *Diuca diuca* under projected future climate scenarios. For 2050 (**A**) and 2070 (**B**), color transitions from cold to warm indicate low to high-risk values, respectively; grey color shows zero-risk. The bar plots show the proportion of pixels for each unique risk index value. Panels (**C**) for 2050 and (**D**) for 2070 show bar plots representing the proportion of pixels for each unique risk index value within protected areas.

Two maps illustrating potential risk areas for *G. cristata* were produced for 2050 and 2070, covering approximately 1,193,233 km^2^ (Fig. 4A-B). From these areas, zero-risk, i.e., regions that are consistently maintained across future climate projections, are expected to cover 24.1% (287,259 km^2^) in 2050 and 20.8% (248,522 km^2^) in 2070. These areas are mainly located where several ecoregions meet: the northeastern portion of the Espinal, Humid Chaco, Southern Cone of Mesopotamia, Humid pampas and in the northwest portion of Uruguayan Savannah. Additionally, two disjunct areas are recovered, one in the west portion of Humid Pampas and in the eastern portion of the Uruguayan Savanna.

On the other hand, risk areas are expected to cover 75.9% (905,974 km^2^) in 2050 and 79.2% (944,711 km^2^) in 2070. Risk index ranged from 3 to 40 (mean = 10.98 ± 7.84) for 2050 and 3 to 31 (mean = 10.24 ± 6.25) for 2070. Risk levels were defined as follows: low risk for values between 3 and 14, moderate risk for values between 15 and 28, and high risk for values between 29 and the maximum risk value for each period, i.e., 40 for 2050 and 31 for 2070. The risk values distribution is right-skewed, being most sites, 81.8% for 2050 and 83.1% for 2070, under low potential risk. By 2050, 13.8% of the area is expected to face moderate risk, increasing to 15.7% by 2070. High potential risk is projected to affect 4.5% in 2050 and decline to 1.2% by 2070. For both time periods, these higher risk areas are predominantly located on the periphery of the Yellow Cardinal’s distribution. The most significantly impacted regions are expected to be Monte and Dry Chaco, followed by the Espinal in the south. In the northeastern region, the area’s most at risk include the Humid Chaco bordering the southern cone of Mesopotamia, and the northeast Uruguayan Savannah.

A total of ninety-seven protected areas overlap at some degree with the estimated risk map. The proportion of protected land potentially affected by some level of risk is projected to be 25.30% by 2050 and 29.62% by 2070. Within these protected areas, the risk index takes values from 3 to 34 in 2050 and 3 to 32 in 2070, covering 3.80% (45,403 km^2^) of the total estimated risk surface (Fig. 4C-D, Table S6). Of the 45,403 km^2^ of at-risk surface within protected areas, 58.6% in 2050 and 44% in 2070 are projected to fall under low-risk category, while 7.7% in 2050 and 33.8% in 2070 are expected to face moderate-risk levels (Fig. 4C-D). High-risk levels are predicted in 4% of PAs in 2050 and only 0.1% in 2070. Additionally, 32.8% in 2050 and 22% in 2070 of the estimated risk areas are expected to remain at zero risk within protected areas (Fig. 4C-D).

We focused on protected areas larger than 100 km^2^ and with at least 40% of their surface overlapping with the estimated risk map. Among these, we identified two key groups: 1) protected areas with at least 80% of their surface classified as zero risk in both scenarios, and 2) protected areas where at least 40% of their surface is projected to experience moderate to high-risk levels (Table S6). Protected areas where over 80% of their surface remains at zero risk in both scenarios include: Rincón de Santa María Natural Reserve, El Gato y Lomas Limpias Multiple-Use Reserve, El Palmar National Park in Argentina, and Montes del Queguay Protected Area with Managed Resources and Esteros de Farrapos e Islas del Río Uruguay National Park in Uruguay. In contrast, protected areas where at least 40% of their surface is projected to face moderate to high risk levels in both scenarios include: Agua Dulce Multiple-Use Reserve, Iberá and Mburucuyá National Parks in Argentina. By 2070, this group expands to include the Lagunas y Esteros del Iberá Ramsar Site-Natural Reserve, Telteca Natural Reserve, and Isla del Cerrito Provincial Reserve in Argentina, as well as Lagoa do Peixe National Park in Brazil.

## Discussion

Abiotic changes and biotic interactions (e.g., new predators, competition, hybridization) are very likely to affect the persistence of small populations in the future^28,29^. In this study, we estimated the geographical distribution of *Gubernatrix cristata,* an endangered bird endemic to South America. This allowed us to describe its environmental suitability and its representation in geographical space. Our model was field validated by a citizen science program that detected Yellow Cardinals in new locations predicted by the model, potentially representing new populations. Future climate projections indicate a southeastern expansion of its current distribution, leading to decreased protection under existing PAs systems. This geographical shift is also expected to increase human impact and intensify interactions with the hybridizing species *Diuca diuca.* By integrating the potential geographic range loss due to climate change and increased contact with *D.* *diuca*, we identified key risk areas, predominantly at the periphery of the cardinal’s distribution. This spatially explicit comprehensive study of *G. cristata* distribution and its main threats provides valuable information for planning effective conservation strategies.

### Two isolated environmentally suitable areas

Understanding the ecological requirements of endangered species is essential for delineating their distributional range limits and thus setting conservation planning priorities^30^. We obtained the first distribution map of the Yellow Cardinal by estimating its environmental suitability, which elucidated its constraints across the entire range using climatic and land cover variables. *Gubernatrix* *cristata* shows greater environmental suitability in areas with lower annual precipitation and precipitation seasonality, higher shrub prevalence, and higher annual temperature coupled with relatively constant temperature, i.e., reduced isothermality. Despite limited information on habitat use and selection by the Yellow Cardinal, the relevance of shrub prevalence for its environmental suitability suggests a preference for shrublands potentially for nesting, as was previously reported^31^. Although traditionally associated with the Espinal, our model identifies the Monte ecoregion, characterized by zygophyllaceas shrubs and open woodlands of *Prosopis* spp^32^, as an important habitat for cardinals. This is supported by the species’ higher abundance in southern Buenos Aires, where the Espinal and Monte ecoregions intersect^12^, and by most known occurrence records being in the Monte ecoregion, as found in Domínguez et al*.*, 2020. Further evidence from various studies supports the association of this species with open habitats characterized by short herbaceous vegetation^7,14,33–36^.

Correlation between environmental variables is an inherent challenge in studies of this sort. It is possible that the variables identified as significant contributors to our model are not the direct drivers of *G. cristata* distribution, but are instead correlated with other, unaccounted factors. Regardless of this, our findings remain valuable for identifying potential habitats and formulating hypotheses about the factors shaping the bird’s realized niche.

Our ecological niche modeling analysis revealed two primary and isolated suitable climatic zones for the Yellow Cardinal: one in the south and another in the northeast of the distribution, consistent with findings by Reales et al. 2019. Despite historical records in the center of the distribution, our model no longer predicts suitable conditions there, corroborated by the absence of occurrences in the citizen science survey. This central area, mainly within the Espinal ecoregion, has undergone extensive agricultural and urban transformation, impacting nearly 49% of its landscape^37^. Soil fertility and proximity to humid pampas have driven extensive land use changes in the Espinal^11^, contributing to habitat loss for the cardinal and pushing its increasing presence into drier shrublands in the southern regions, such as those in the Monte ecoregion, as our model predicts and surveys support^11,12^. In contrast, only 4% of the Monte ecoregion has been transformed, mainly due to livestock production and biological invasions^37^. The northeastern patch encompasses several threatened ecoregions, including the Parana flooded, Espinal, Southern Cone of Mesopotamian and Uruguayan savannas, Humid Chaco and Humid Pampas. These areas face threats from intensive deforestation, drainage for agriculture, urbanization, logging, and cycles of drought-fire^38^ potentially impacting the suitability for Yellow Cardinal populations. Comparative studies on cardinal occurrences and land use changes over time in these regions could further substantiate these insights.

Assessing the environmental suitability for the Yellow Cardinal using species distribution models reveals critical insights into its geographical distribution and environmental adaptability. On the one hand, the few records outside the predicted suitable area (in central Argentina and Uruguay) may be attributed to escaped birds from illegal pet trade or to limitations in the models, which potentially did not consider other influential factors besides climate and land use. Alternatively, this could also indicate the species’ phenotypic plasticity and adaptability to new environments. Recognizing the adaptive potential of the Yellow Cardinal is extremely important for assessing its extinction risk, although this requires further long-term studies. On the other hand, many regions identified as currently suitable do not host any known populations. This apparent discrepancy might be due to several nonexclusive hypotheses, including insufficient surveys that have failed to detect undiscovered populations, illegal capture, oversimplified models, or negative biotic interactions.

The acquisition of an independent dataset through a citizen science survey helped validate the model at more than one hundred points, making the accuracy of our model reliable. While standardized citizen science data offers a useful alternative to acquiring large-scale external datasets, it may be biased toward areas of higher observer density^39^. Despite potential biases, which can be mitigated through additional sampling, citizen science is still a useful method for surveying at regional scales, as demonstrated in this study. Furthermore, collaboration with non-governmental organizations promotes valuable scientist-volunteer interactions, enhancing the development of distribution models. Notably, twenty-one of these records not only confirm the model-predicted areas but also reveal new, potentially isolated populations more than 15 km from the nearest known populations.

### Significant geographic shifts predicted in climate change scenarios

Our analysis predicts significant geographic range shifts under various climate change scenarios, which are expected to intensify by 2070. Notably, the geographic range loss is anticipated to be more severe by 2050 than by 2070. However, the extent of geographic range preserved by 2070 is expected to be less than that in 2050, although this reduction is somewhat mitigated by the emergence of new suitable areas. Furthermore, the variation among climate scenarios in terms of geographic range gains and losses is much greater for 2070 than for 2050. This greater variability in 2070, already noted in other studies^5^, highlights the complexity of formulating climate change mitigation policies based solely on average projections.

Moreover, the projected increase in geographic range (reaching a maximum under RCP 8.5) is accompanied by a gradual shift to higher latitudes and eastern regions. This shift to the south aligns with trends reported for other bird species (review in^40,41^). Specifically, climate change projections for endangered birds, including *G. cristata,* in subtropical temperate grasslands in Argentina, indicate a similar pattern, with suitable areas shifting from north to south and from west to east^42^. To take advantage of these new suitable geographic areas, cardinals should disperse, a critical and risky event that can reduce survival, as observed in other dispersing birds. Cardinals face challenges as they are non-migratory, territorial, and males tend to exhibit philopatry^14^. However, Brazilian cardinal populations were reported to have relatively large breeding territories (mean = 7.9 ± 5.6 ha) and home range sizes (mean = 27.7 ± 9.1 ha^14^, suggesting a potential dispersal capacity at least during the breeding season.

The greatest shift projected for 2070 in RCP 8.5 includes expansion into Humid Pampas, Uruguayan Savannas and Monte ecoregions. However, the Humid Pampas, as the most transformed ecoregion of Argentina, has nearly 78% of its surface intended for agriculture, commercial livestock production, and urbanization^37^, while the Uruguayan Savanna is critically endangered mainly by cattle ranching and agriculture, with 80% of Uruguayan territory used for such purposes^43^. These conditions pose significant challenges for cardinal dispersal due to limited available habitat. Moreover, the development of the essential phytogeography necessary for nesting could lag behind climatic changes, further affecting cardinal dispersal. Even if species can disperse quickly enough to match suitable climates, they often encounter novel abiotic and biotic conditions that complicate adaptation^44^. Cardinal’s low genetic diversity^9^ might limit their adaptive potential^45^, making adaptation to these novel conditions less likely. It is also unlikely that populations in areas predicted to lose geographic range will be able to relocate to nearby suitable areas, thus increasing the risk of extinction. Although between 53 and 65% of environmental suitability is expected to remain geographically represented under future climatic conditions, serving as potential refuges for cardinal populations, the projection for 2070 in the worst-case scenario shows that only 35% of environmental suitability would be geographically represented. Considering that macroclimate variables can overestimate range shifts, microclimate conditions could play a crucial role in estimating localized climate change effects^46^. These effects could lead to population fragmentation, alterations in demography, and changes in community composition and ecosystem function^46^. Moreover, Yellow Cardinal populations inhabiting shrublands and open woodlands might be particularly sensitive to warming in these microclimates, as the amplified effects of solar irradiance on near-ground temperatures are especially pronounced in such habitats^46,47^, as the study case. Continuous monitoring at macro and microclimate level cardinal populations might help us to understand if the species is able to sustain itself in these areas or even expand its range under these conditions.

### Predicted shift to more anthropized and less protected areas

We specifically evaluated the effects of human pressure on the current and potential distributions of Yellow Cardinals, as illegal capture is a major threat to this species. Our findings suggest that 4.5% of the species’ current distribution coincides with areas of high HFP. This overlap might account for the absence of records in regions our model identified as suitable, such as North of La Pampa Province, northwest of Corrientes Province, and Rio Grande do Sul. Indeed, Reales et al. (2023) noted that cardinals tend to inhabit areas distant from permanent roads, likely as a response to capture pressures near these routes. This avoidance pattern could intensify under future climate conditions, potentially deterring the species from migrating to such areas. In fact, our model projects an increase in the overlap between future projections of *G.* *cristata*’s distribution and areas of high HFP under most future climate change scenarios. While the current human footprint may not be representative of future scenarios, particularly those extending to 2070, it serves as a basis for considering how climate change could impact the species if the level of anthropization remains at least at its present level. Protected areas could mitigate these negative anthropogenic impacts, given that Yellow Cardinal populations in PAs are safeguarded at least against illegal capture and habitat transformation. However, the species is currently underrepresented (< 5% of its range) in the current system of PAs, and unfortunately, its future representation is expected to decrease under most climate change scenarios. Moreover, current human footprint models do not account for future land use changes, which could further exacerbate habitat loss and fragmentation. Considering the predicted increase in the Yellow Cardinal’s EGD, a maintained or decreased overlap with reserves indicates a shift in distribution toward regions where no PAs exist. This suggests that the current PA system may not only be insufficient under present conditions but may also fail to protect the species from increasing land-use pressures in the future. Expanding the PAs network can be beneficial for the Yellow Cardinal and other species, and our results can help identify where the establishment of new PAs would be most effective in mitigating both current and future threats.

### Hybridization risk and other biotic stressors

As a negative biotic interaction, we focused on the overlapping distribution of the Yellow Cardinal and its sister species the Common Diuca Finch during the breeding season. Previous reports^19^ have highlighted instances of hybridization between these species, posing a significant threat to the persistence of Yellow Cardinal populations. In threatened species, hybridization poses a critical risk by reducing effective population size^21^ and, in cases of genetic introgression, by compromising genome integrity through increased mutational load. Introgression has been linked to negative effects, such as the genetic erosion of endangered populations potentially leading to extinction^48^. Our analysis suggests that the geographic overlap between the EGDs of both species is currently 11.65%, with slight variations in the future projections. Interestingly, the predicted geographic range of the Common Diuca Finch is expected to experience a modest reduction under most future climate change scenarios, which would be unlikely to affect the persistence of such widely distributed, non-threatened species.

As other negative biotic interactions can affect the Yellow Cardinal’s fitness and pose serious threats across its geographic range, it would be advisable to evaluate them spatio-temporally in the context of climate change. Such interactions include brood parasitism by shiny cowbirds (*Molothrus bonariensis*), which reduces reproductive success through increased chick competition and egg damage, and parasitism by *Philornis* spp*.* flies, causing myiasis in nestlings^33,49–51^. Although both biotic interactions are documented threats to the Yellow Cardinal, uncertainties regarding the identity of *Philornis* species involved and the complex environmental constraints on *Molothrus bonariensis*, a widely distributed generalist species, prevented their inclusion in our modeling approach.

### Conservation implications and risk map

Our study highlights the considerable potential impact of climate change, ecological interactions, and anthropogenic stressors on the Yellow Cardinal. However, the future resilience of this species to such pressures will depend not only on the availability of suitable niches, but also on its adaptive potential and phenotypic plasticity. While our model offers an initial assessment of these potential impacts, it provides valuable information for future conservation planning. We project a large geographic shift in the suitable areas for the Yellow Cardinal, regrettably toward areas with a higher degree of anthropic pressures and diminished protection status. The risk maps produced in this study reveal the areas predicted to face a loss of geographic range, as well as those threatened by potential hybridization with the sister species Common Diuca Finch. The areas of higher potential risk are mainly at the edges of the cardinal distribution, notably at the northern and western margins. Despite the predicted range expansion in the opposite direction (toward the east and south), the loss of these marginal populations could result in a reduced gene pool for the species. Our results raise concerns about the possible permanent loss of genetic variation unique to the genetic cluster known as management unit 2, which is significant given the already low genetic variation and population differentiation previously reported^9^.

It was our motivation to provide supportive information to be used in the management plan of the species to help ensure its persistence. The information provided here can assist in selecting the best areas for bird releases in restocking or reintroduction programs. These areas are identified by our model as 1. environmentally suitable both now and in the future, 2. characterized by minimal biotic pressures (low hybridization likelihood), and 3. minimal anthropic pressures (areas already under protection or with low HFP). We also highlight the vulnerability of the current protected area system, which may face increasing levels of moderate potential risk while zero-risk areas continue to decline. Although the maximum risk index is projected to be higher in 2050 (40) than in 2070 (31), the total area at risk is expected to be larger in 2070, primarily due to an increase in moderate-risk levels. Moreover, zero-risk areas within PAs are expected to decrease significantly by 2070 compared to 2050. These findings indicate that Yellow Cardinals are threatened both by geographic range loss and by potential hybridization with *Diuca diuca* driven by climate change at the species’ marginal distribution and within protected areas. Regarding zero-risk areas, a large continuous surface patch is located in a heterogenous region where four ecoregions converge. These areas could be valuable for an in-situ conservation strategy for Yellow Cardinals, as their high environmental heterogeneity and long-term stability are key factors for sustaining viable populations. Redesigning the current protected area system into a more efficient conservation network, such as increasing connectivity, could enhance biodiversity conservation on a broader scale. This includes linking zero-risk areas like Rincón de Santa María Natural Reserve, El Gato y Lomas Limpias Multiple-Use Reserve, and El Palmar National Park in Argentina, as well as Montes del Queguay Protected Area with Managed Resources and Esteros de Farrapos e Islas del Río Uruguay National Park in Uruguay, which are currently far apart from each other by approximately 80 km. Since these zero-risk areas form a mosaic of ecoregions, they encompass both high macro-and microclimate heterogeneity, which could mitigate the impacts of climate change on other biotic components, as previously demonstrated^46^. This study is the first to summarize several potential threats to the Yellow Cardinal using geographically explicit information. However, further studies monitoring natural populations and a comprehensive understanding of the drivers of population declines are essential, as mitigating these factors is crucial for the species’ continued survival. Our findings demonstrate how a conservation gap analysis can guide the establishment of new PAs with focus on the management of an emblematic species at a large scale. Based on these results, we propose an integrated management approach that targets the key gaps we have identified.

## Methods

### Occurrence records

We compiled 758 occurrence records of *Gubernatrix cristata* from various sources, spanning from 1953 to 2018: field campaigns performed in Argentina^9,52^; Argentinean ornithological collections FML, MACN and MLP (museum abbreviation follows^53^); the eBird database (https://ebird.org/home, downloaded in February 2018); a citizen science program focused on *G. cristata* organized by the NGO Aves Argentinas ^11^ and a database created by the Ministry of Environment and Sustainable Development of Argentina. For Brazil, we included records from Beier et al. (2017), and for Uruguay, records provided by O. Blumetto and A. Ricchetto (personal communication, 2014, Table 1). After removing duplicate records, we applied a 10 km spatial filter to reduce spatial autocorrelation among presence points, retaining 324 records. These were then evenly split (50% for testing and 50% for training) to develop the ecological niche model using package *ellipsenm* v. 0.3.4^54^ in R^55^. We used 50% of the records for training after confirming that this subset adequately represents the full range of environmental conditions captured by the complete dataset.

**Table 1:** Data sources of Yellow Cardinal’s occurrence records considered in this study.

Source category	Number of occurrence records	Temporal cover	Source details
Field surveys	30	2012—2013, 2017	Domínguez et al. 2017, Fracas et al. 2023
Museum Collections	23	1953—1962	Museo Argentino de Ciencias Naturales, Museo de La Plata, Museo Miguel Lillo de Ciencias Naturales
Scientific Articles	1	2017	Beier et al. 2017
Free online databases	451	1986—1987	eBird
Government organization database	138	1963—1983, 1985—1998, 1996, 1998, 2011–2013	Ministry of Environment and Sustainable Development of Argentina
Non-government organization database	102	2015—2017	Domínguez et al. 2020, Aves Argentinas
Specialist birdwatchers	13	1984, 2003—2005, 2008, 2010—2012	Oscar Blumetto, Alvaro Ricchetto

### Estimations of geographic distributions and projections on future climate scenarios

We estimated the geographical distribution of *G. cristata* using ecological niche modeling (ENM) protocols. ENMs combine species presence records with local environmental variables, providing an estimation of environmental suitability^56^. To adequately model the species’ niche, we adopted the Biotic-Abiotic-Mobility theoretical framework^56^. A biotic component was considered in post-processing modeling, particularly concerning biotic interactions like hybridization because the interacting species could affect each other and thus cannot be considered as scenopoetic variables^57^. For the mobility component, we designed a calibration area a priori^58^ considering ecoregions (sensu.^59^) with known species presence. Regarding the abiotic component, we included climatic and land use variables in our models. The macrophysiology of endotherms is constrained by temperature and water balance, which impact assimilation, metabolism, and activity rates. Moreover, land vegetation type is a key factor for Yellow Cardinal distribution because it can impact on nesting sites and food resource availability. We used 15 bioclimatic variables based on an interpolation of observed data, representative of 1950–2000 (WorldClim v1.4)^60^, excluding four variables (bio 8, 9, 18, 19) due to known artifacts ^61^ and two variables of vegetation prevalence: classes 4 (Mixed/Other Trees) and 5 (Shrubs) based on satellite information from 1992 to 2006 (EarthEnv Global 1-km Consensus Land Cover v1)^62^. All variables were at a 30-s spatial resolution (≈ 1 × 1 km per pixel at the equator) and were clipped to the calibration area. Among the bioclimatic variables, we detected and excluded highly correlated variables (r ≥ 0.8, Fig. S5) through Pearson correlations performed in the *ntbox* v. 0.7.1 R package^63^. We then retained the variable that contributed the most to our models through a jackknife analysis in Maxent v3.3.3 K^64^. Finally, six bioclimatic variables were selected to perform ecological niche modeling (Fig. S2).

We calibrated and selected the best ENMs using Maxent (maximum entropy method) through the R package *kuenm* v. 1.1.10^65^. The candidate models were obtained by all possible combinations among four regularization multipliers (0.1, 0.5, 1, 2) and five types of feature classes (linear = l, quadratic = q, product = p, threshold = t, and hinge = h). The best models were selected based on strong statistical significance (partial ROC analysis across 100 bootstrapping iterations using half of the data), omission rates ≤ 5%, and among these, those with delta AICc values ≤ 2. We created the final model using the complete dataset of occurrences and the selected parameterizations, tested through 5 bootstrap trials producing logistic outputs. Evaluations of the final model consisted of calculating partial ROC and omission rates (based on error = 5%) using an independent dataset obtained through a citizen science program (see Sect. “Field validation of estimated geographic distribution”)

The final model was transferred to four future representative concentration pathways (RCPs 2.6, 4.5, 6.0, and 8.5 for 2050 and 2070) in South America. We used the ‘GCM compareR’ web application^66^ to identify the most appropriate Global Circulation Models (GCMs) from the Coupled Model Intercomparison Project 5^67^. This allowed us to select a model whose forecasts closely matched the mean projections across all evaluated GCMs, thereby minimizing the uncertainty from outlier projections. Specifically, we compared forecasts of two bioclimatic variables (mean annual temperature and annual precipitation) within the known distribution area of *G. cristata*, identifying the HadGEM2-AO model as the most suitable for our analysis. While vegetation is undoubtedly influenced by climate conditions, we aimed to isolate the effect of climate change alone by controlling vegetation. For this reason, we kept the current land cover variables constant in our future projections.

To generate binary presence-absence maps for both current and future projections, we applied a modified lower presence threshold. This adjustment accounts for potential errors in presence data, e.g., misidentifications, or occasional sightings of Yellow Cardinals that have escaped captivity^68^. This threshold included 100% of the presence points, deducting a 20% dataset error^69^ based on our experience in collecting the presence data. This conservative method minimizes the commission error rate. Moreover, future potential EGDs were cropped to the calibration area, thus excluding potentially spurious areas produced by extrapolation during the model transfer process.

### Field validation of estimated geographic distribution

To verify the accuracy of our model and to locate previously unknown populations, field validation of the Yellow Cardinal’s EGD was conducted. This is recommended when ENMs are used to obtain suitable areas for species whose total distribution and habitat preferences are only partially known, such as for our species of study^70^. A citizen science program coordinated by NGO Aves Argentinas carried out this field validation in 2018, focusing on Argentina, where Yellow Cardinals are mainly distributed. Based on the ENM obtained, we proposed target survey areas characterized by suitable environments yet lacking previous presence records. The survey design and data processing are described in Domínguez et al., 2020. To validate the EGD, we counted new yellow cardinals’ sightings within these predicted areas.

### Potential impact of climate change on range size and geographic shift

To evaluate the effect of climate on suitable Yellow Cardinal areas, we measured variations in the predicted range size. Specifically, we assessed the extent to which geographic range was lost, gained, or maintained relative to the current conditions. Additionally, we quantified distributional shifts by calculating geographic displacement through the overlap index^71^ as follows:

1 $$O=nf\cap{p}/nf\cup{p}$$

where n is the number of cells, and f ∩ p and f ∪ p are the regions intersected and combined (sum) between the present (p) and future (f) distributions, respectively.

### Quantifying anthropic pressures, state of protection, and biotic threats in current and future suitable areas

To investigate the level of anthropic pressure, we obtained areas with high impact of Human Footprint index from the NASA Data Center at 1 km resolution scale^72^ (Supplementary text S1). Then, we calculated the percentage of overlap between these areas and the current and future predicted ranges of the Yellow Cardinal.

To explore the degree of protection of the Yellow Cardinal, we quantified the proportion of its current and future predicted range that falls within protected areas. The layer of the current PAs system was obtained by combining information from the *World Database on Protected Areas*^73^), the *Ministerio de Vivienda y Ordenamiento Territorial y Medio Ambiente* of Uruguay (https://www.dinama.gub.uy/geoservicios/), UNESCO biosphere reserves, and Ramsar sites, due to their importance for birds locally (Table S5, Supplementary text S2).

To assess local biotic threats, we estimated the potential contact zone between the EGDs of *G. cristata* and *D.* *diuca* by measuring their current and future geographic overlap. We obtained *D.* *diuca’s* EGD for current and future climate scenarios using methods similar to those used for *G. cristata*, with species-specific modifications detailed in Supplementary text S3.

### Inference of risk maps for the yellow cardinal

To identify areas of potential risk for *Gubernatrix cristata*, we created risk maps for the years 2050 and 2070, combining both the potential loss of geographic range and the potential hybridization contact areas with *Diuca diuca* under future climate scenarios. For this, we created two raster layers, i.e., potential geographic range loss and potential hybridization contact area, for each period, where higher cell values suggest that both geographic range loss and contact zones are predicted in all scenarios. Then, we calculated the potential risk areas for each period by applying a weighted sum, assigning a weight of 0.7 for potential geographic range loss and 0.3 for potential hybridization contact areas. This approach was designed to appropriately reflect the relative importance of each factor in determining the overall extinction risk. Additionally, we included a zero-risk term, defined as areas consistently maintained across all scenarios for each period, with no potential contact with *Diuca diuca*. (Supplementary Text S4). Finally, we quantified the proportion of potential risk areas and zero-risk areas within the current system of protected areas (see layer acquisition in the section above). Geoprocessing and map editing were conducted using ArcGIS Pro 3.2.2 (Esri).

## Supplementary Information

Supplementary Information 1.

Supplementary Information 2.

Supplementary Information 3.

Supplementary Information 4.

Supplementary Information 5.

Supplementary Information 6.

Supplementary Information 7.

Supplementary Information 8.

## Data Availability

The data and R code for our analyses are available at a Mendeley repository (10.17632/t4hjn28b2s.2). While we do not publicly disclose sensitive occurrence data points for the endangered species to protect their locations, we will make these data available upon request for purposes of reproducibility (contact R.M.: regina.g.medina@gmail.com).
