# Supplementary material for: Vulnerability of Gubernatrix cristata to climate change, anthropogenic pressures, and hybridization threats
Source: Sci Rep. 2025 Apr 9;15:12152. doi: 10.1038/s41598-025-94293-7 (PMC11982183; doi:10.1038/s41598-025-94293-7)
Supplement: Supplementary file 1 — Supplementary Information 1. [file 41598_2025_94293_MOESM1_ESM.docx]

**Supplementary information**

**Assessing the vulnerability of the Yellow Cardinal (*Gubernatrix cristata*) to climate change, anthropogenic pressures, and hybridization threats**

Regina Gabriela Medina & Marisol Domínguez


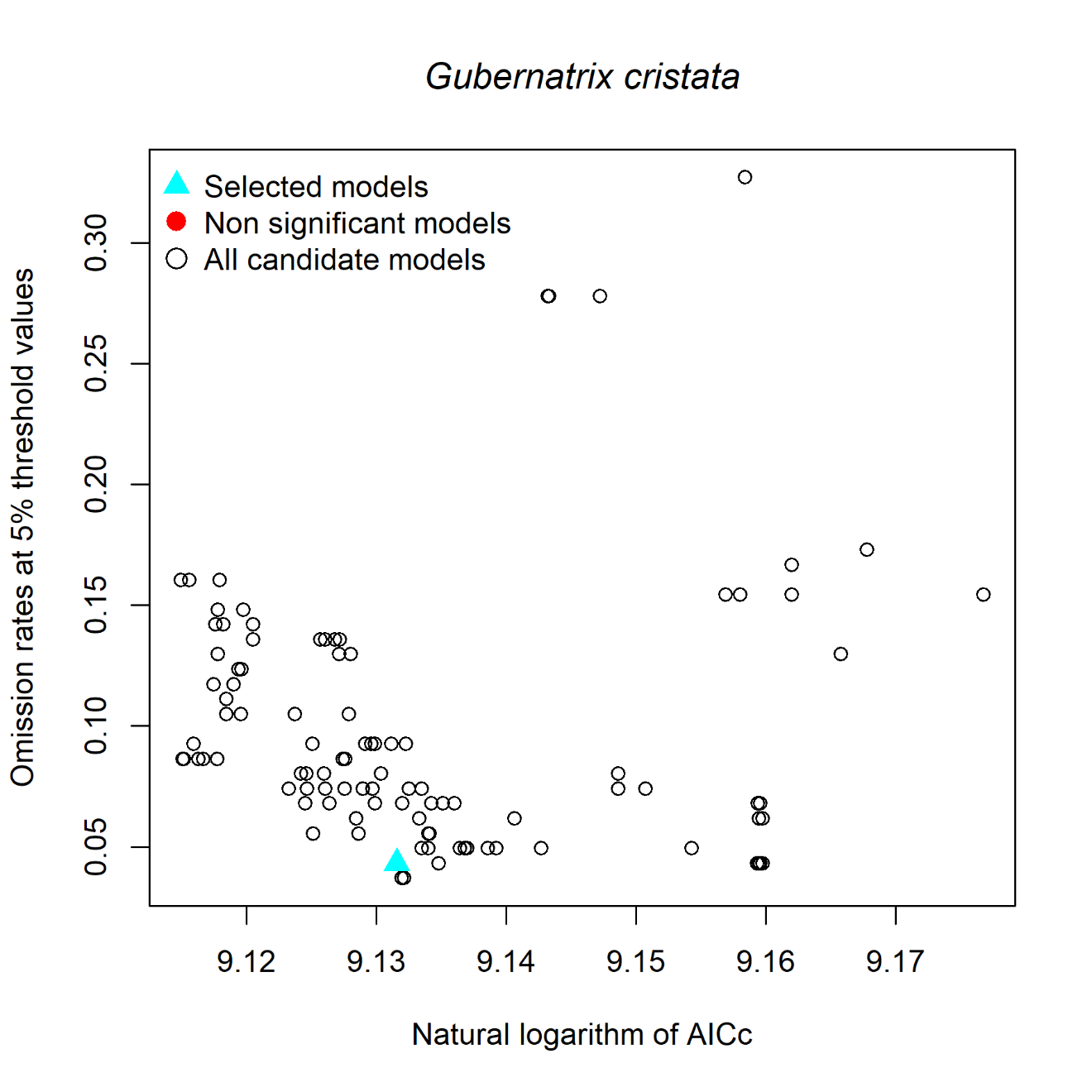


**Figure S1**. Model’s performance for *Gubernatrix cristata*, displaying the distribution of all models (empty grey circles), non-significant models (red circles), and selected models (filled light-blue triangles) in terms of omission rates and AICc.


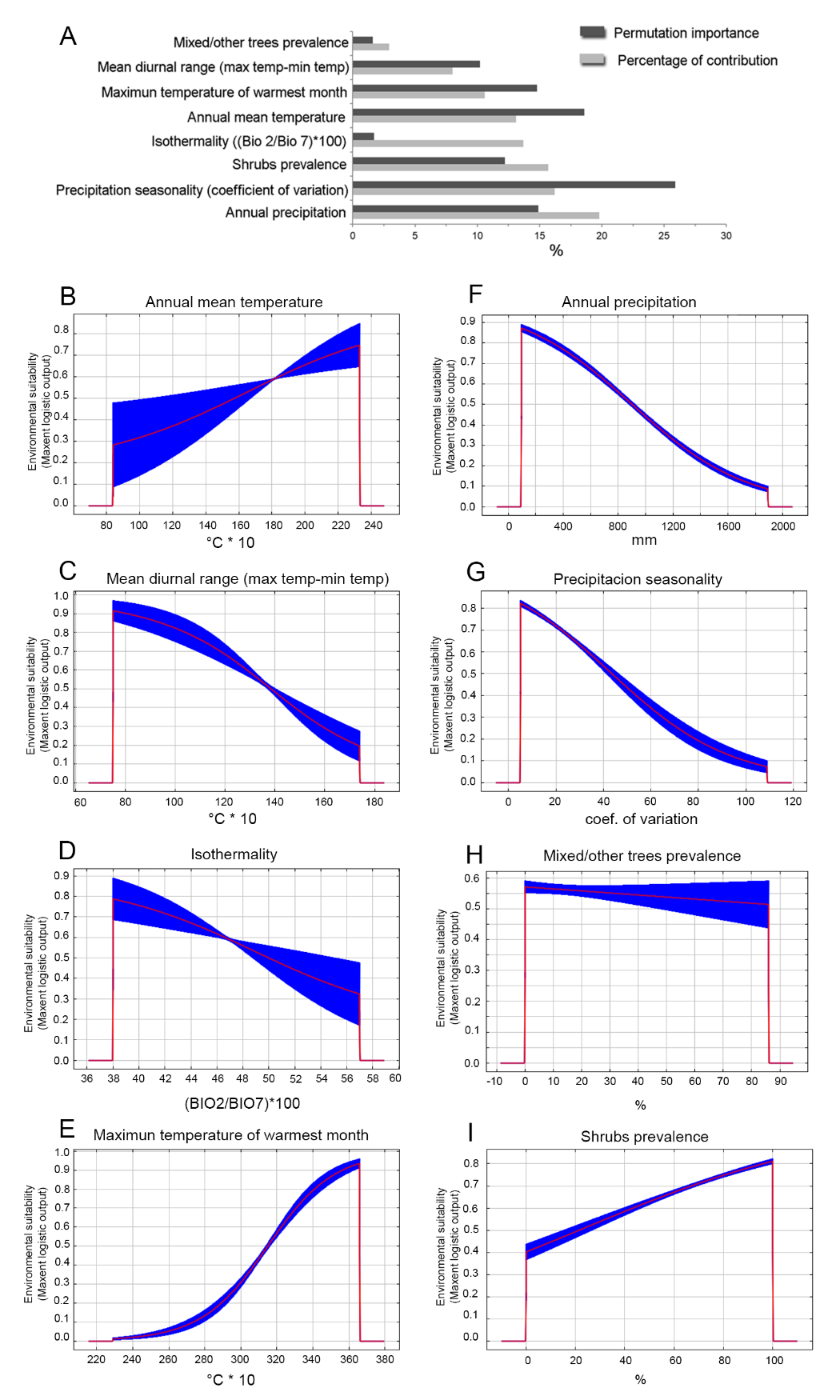


**Figure S2**. A. Environmental variables used in the ecological niche modelling of *G. cristata*. A. Percentage of contribution and permutation importance of each environmental variable used in the ENM. B-I. Response curves of each variable in the Maxent model for environmental suitability. Red lines indicate the mean values, while blue areas denote one standard deviation limit, resulting from bootstrap replicates in model runs.


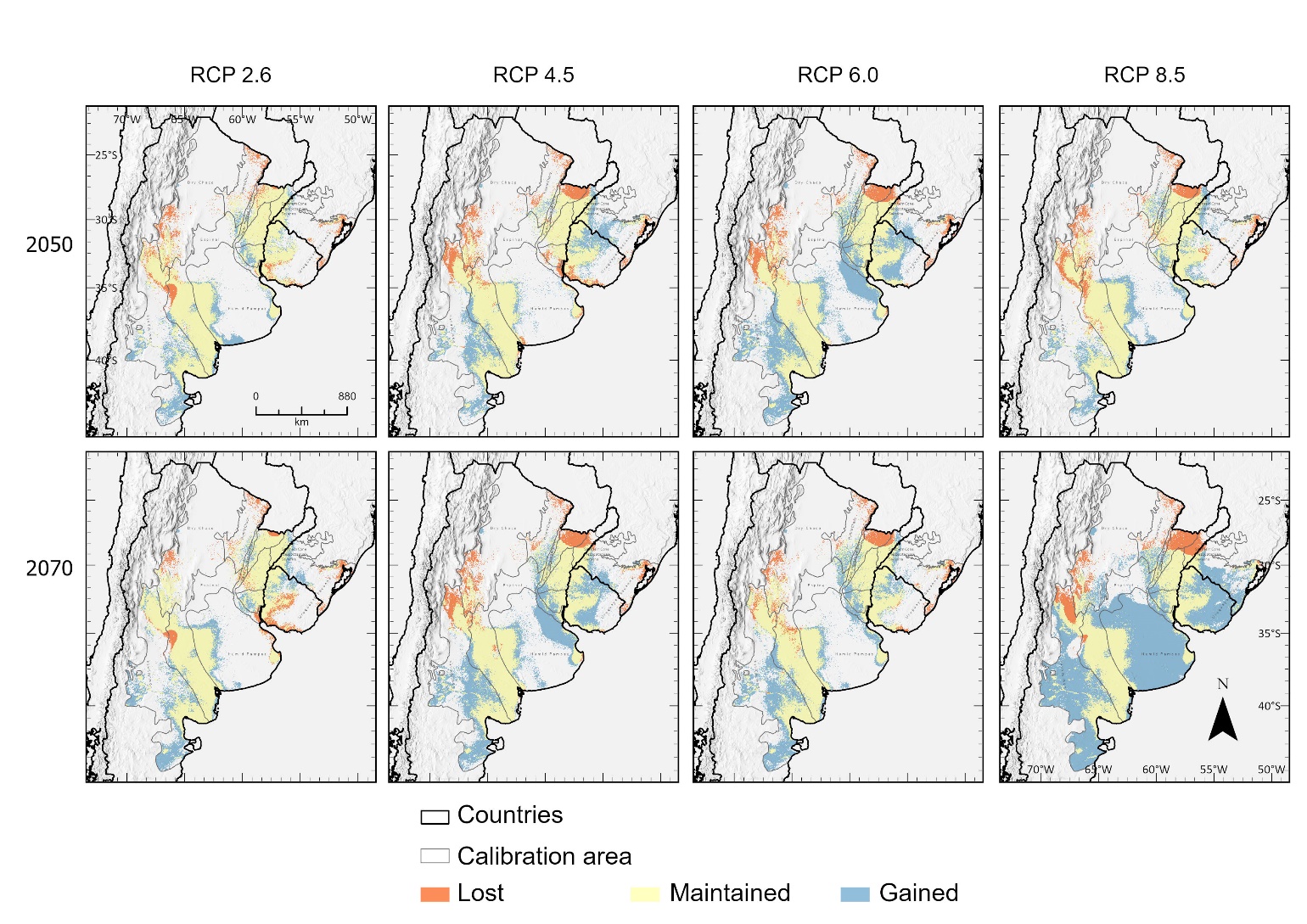


**Figure S3.** Predicted changes in *G. cristata*’s EGD. Projected geographic distribution of *G. cristata* under all climate scenarios for 2050 and 2070. Blue, orange and yellow colors represent gained, lost and maintained areas by *G. cristata* comparing the current scenario with each projected future scenario.


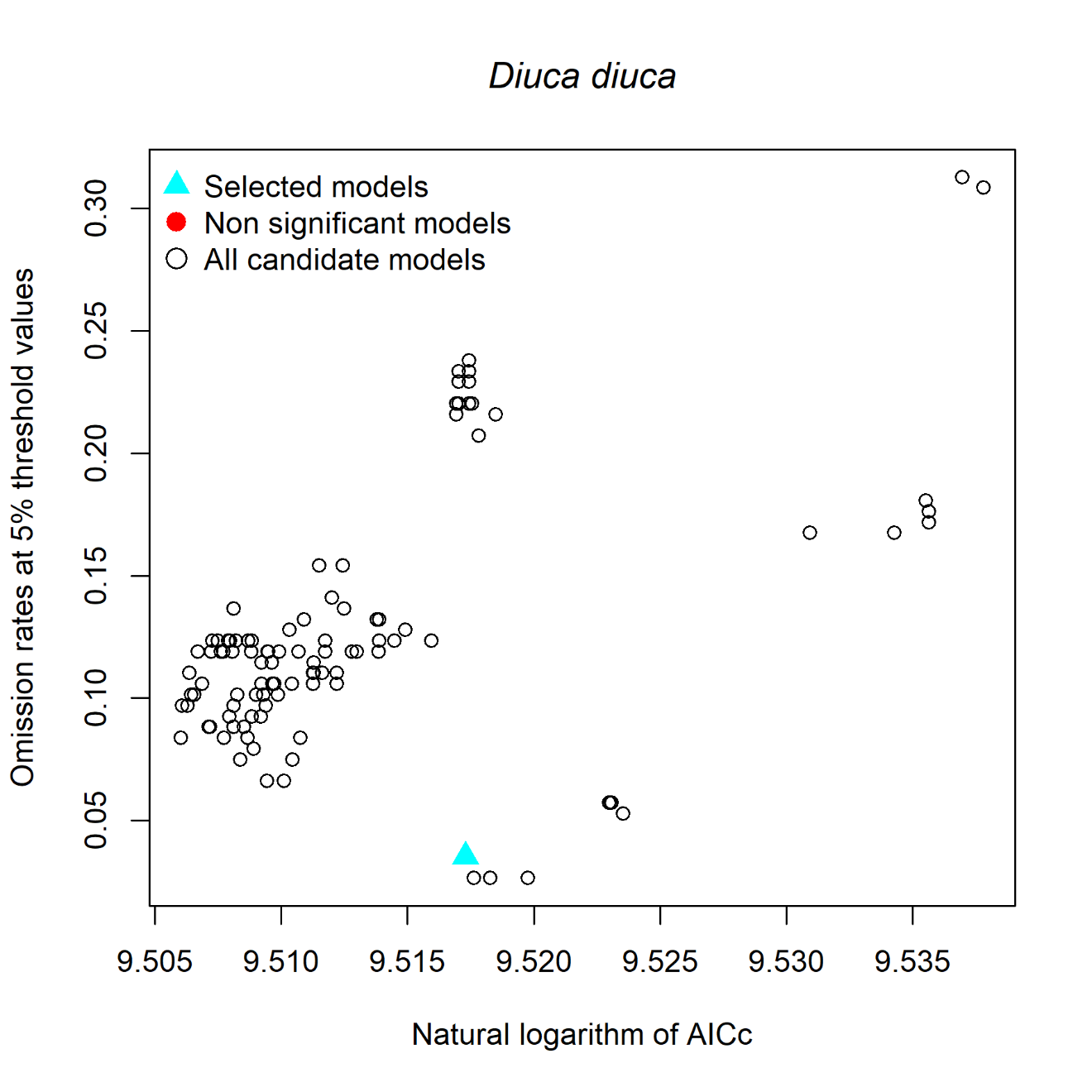


**Figure S4.** Model’s performance for *Diuca diuca*, displaying the distribution of all models (empty grey circles), non-significant models (red circles), and selected models (filled light-blue triangles) in terms of omission rates and AICc.


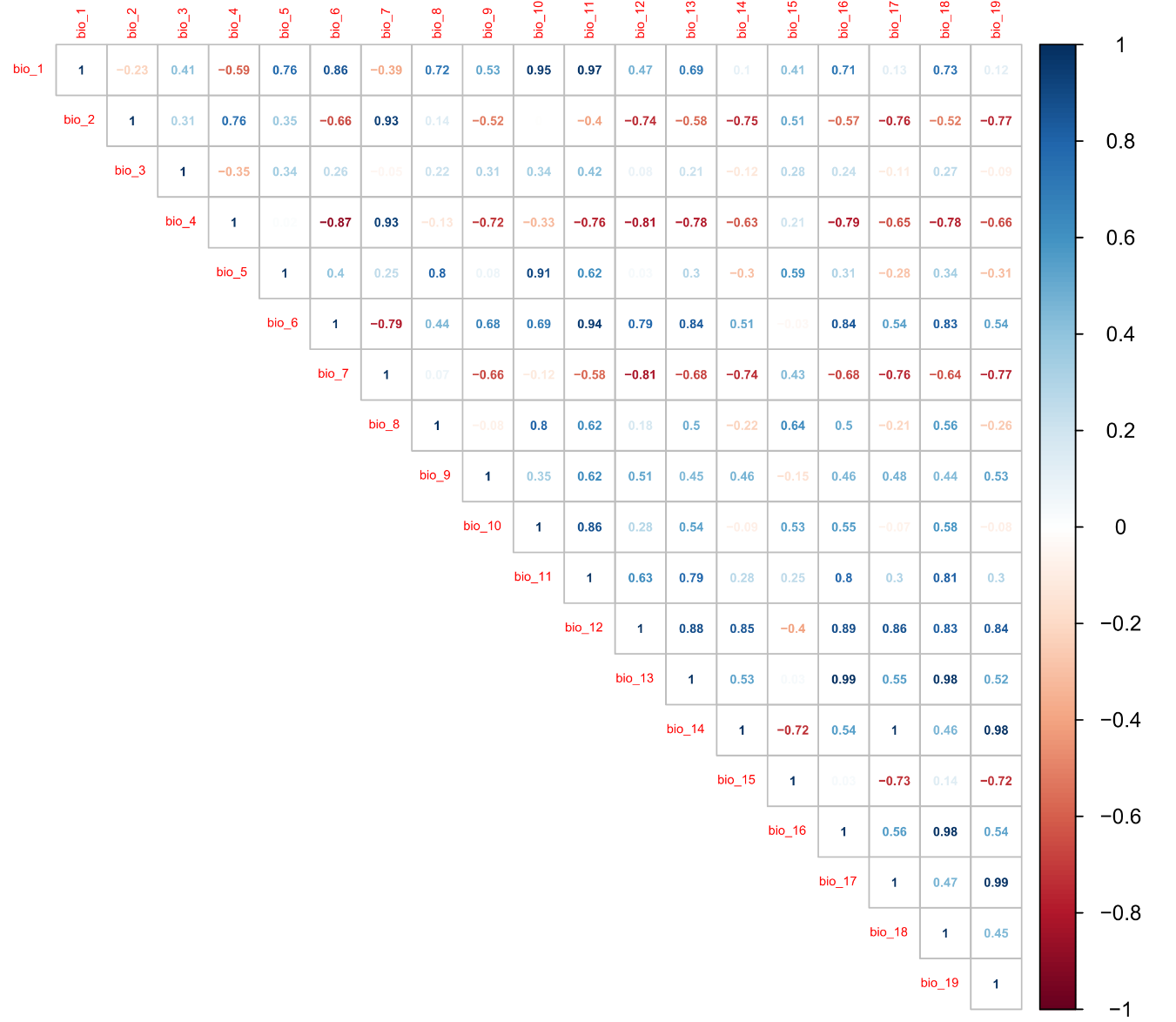


**Figure S5.** Pearson correlations between climatic variables used for the estimations of the geographical distribution of *G. cristata* using ecological niche modeling.
