# Supplementary material for: Vulnerability of Gubernatrix cristata to climate change, anthropogenic pressures, and hybridization threats
Source: Sci Rep. 2025 Apr 9;15:12152. doi: 10.1038/s41598-025-94293-7 (PMC11982183; doi:10.1038/s41598-025-94293-7)
Supplement: Supplementary file 3 — Supplementary Information 3. [file 41598_2025_94293_MOESM3_ESM.docx]

**Supplementary information**

**Assessing the vulnerability of the Yellow Cardinal (*Gubernatrix cristata*) to climate change, anthropogenic pressures, and hybridization threats**

Regina Gabriela Medina & Marisol Domínguez

**Table S1**. Statistics for models meeting each evaluation criterion.

| Criteria | Number of *G. cristata*´s models | Number of *D. diuca*’s models |
| --- | --- | --- |
| All candidate models | 116 | 124 |
| Statistically significant models | 116 | 124 |
| Models meeting omission rate criteria | 17 | 4 |
| Models meeting AICc criteria | 4 | 2 |
| Statistically significant models meeting omission rate criteria | 17 | 4 |
| Statistically significant models meeting AICc criteria | 4 | 2 |
| Statistically significant models meeting omission rate and AICc criteria | 1 | 1 |
