# Supplementary material for: Vulnerability of Gubernatrix cristata to climate change, anthropogenic pressures, and hybridization threats
Source: Sci Rep. 2025 Apr 9;15:12152. doi: 10.1038/s41598-025-94293-7 (PMC11982183; doi:10.1038/s41598-025-94293-7)
Supplement: Supplementary file 4 — Supplementary Information 4. [file 41598_2025_94293_MOESM4_ESM.docx]

**Supplementary information**

**Assessing the vulnerability of the Yellow Cardinal (*Gubernatrix cristata*) to climate change, anthropogenic pressures, and hybridization threats**

Regina Gabriela Medina & Marisol Domínguez

**Table S3**. Selected model based on the user-specified criteria.

| **Species** | **Model regularization multiplier** | **Feature model** | **Mean AUC ratio** | **pval**  **pROC** | **Omission rate at 5%** | **AICc** | **Delta AICc** | **W AICc** | **Number of parameters** |
| --- | --- | --- | --- | --- | --- | --- | --- | --- | --- |
| ***G. cristata*** | 0.1 | Linear  Product | 1.28 | 0 | 0.04 | 9242.31 | 0 | 1 | 31 |
| ***D. diuca*** | 0.1 | Linear | 1.1 | 0 | 0.04 | 13592.79 | 0 | 1 | 8 |
