# Supplementary material for: Vulnerability of Gubernatrix cristata to climate change, anthropogenic pressures, and hybridization threats
Source: Sci Rep. 2025 Apr 9;15:12152. doi: 10.1038/s41598-025-94293-7 (PMC11982183; doi:10.1038/s41598-025-94293-7)
Supplement: Supplementary file 5 — Supplementary Information 5. [file 41598_2025_94293_MOESM5_ESM.docx]

**Supplementary information**

**Assessing the vulnerability of the Yellow Cardinal (*Gubernatrix cristata*) to climate change, anthropogenic pressures, and hybridization threats**

Regina Gabriela Medina & Marisol Domínguez

**Table S4**. Final model evaluation of *G. cristata* using an independent dataset obtained through a citizen science program.

| **Model regularization multiplier** | **Feature model** | **Mean AUC ratio** | **Partial ROC** | **Omission rate at 5%** |
| --- | --- | --- | --- | --- |
| 0.1 | Linear  Product | 1.51697896 | 0 | 0 |
