# Supplementary material for: Vulnerability of Gubernatrix cristata to climate change, anthropogenic pressures, and hybridization threats
Source: Sci Rep. 2025 Apr 9;15:12152. doi: 10.1038/s41598-025-94293-7 (PMC11982183; doi:10.1038/s41598-025-94293-7)
Supplement: Supplementary file 6 — Supplementary Information 6. [file 41598_2025_94293_MOESM6_ESM.docx]

**Supplementary information**

**Assessing the vulnerability of the Yellow Cardinal (*Gubernatrix cristata*) to climate change, anthropogenic pressures, and hybridization threats**

Regina Gabriela Medina & Marisol Domínguez

**Table S5**. Protected areas of local importance not categorized by IUCN used in the analysis. Designation category, type, year, and area in km^2^ are reported.

| **Name** | **Designation** | **Designation type** | **Reported Area (km^2^)** | **Year** |
| --- | --- | --- | --- | --- |
| Lote 11 Ñacuñan | Forest Reserve | National | 76.82 | 1961 |
| Punta Bermeja | Protected Nature Area | National | 19 | 1971 |
| Los Glaciares National Park | World Heritage Site | International | 4459 | 1981 |
| Iguaçu National Park | World Heritage Site | International | 1700.86 | 1986 |
| Ñacuñan | UNESCO-MAB Biosphere Reserve | International | 122.71 | 1986 |
| Parque Nacional Laguna Blanca | Ramsar Site | International | 112.5 | 1992 |
| Parque Nacional Rio Pilcomayo | Ramsar Site | International | 518.89 | 1992 |
| Reserva Provincial Laguna de Llancanelo | Ramsar Site | International | 650 | 1995 |
| Yabotí | UNESCO-MAB Biosphere Reserve | International | 2537.73 | 1995 |
| Bahía de Samborombón | Ramsar Site | International | 2439.65 | 1997 |
| Villavicencio | Private Reserve | National | 620 | 2000 |
| Bañados del Río Dulce y Laguna de Mar Chiquita | Ramsar Site | International | 9960 | 2002 |
| Lagunas y Esteros del Iberá | Ramsar Site | International | 245.5 | 2002 |
| Las Yungas | UNESCO-MAB Biosphere Reserve | International | 13287.2 | 2002 |
| Monte Loayza | Provincial Reserve | National | 17.4 | 2004 |
| Bañado la Estrella | Provincial Nature Reserve | National | 3816 | 2005 |
| Costanera Sur | Ramsar Site | International | 3.5 | 2005 |
| Parque Provincial El Tromen | Ramsar Site |  | 300 | 2006 |
| Reserva Natural Otamendi | Ramsar Site | International | 30 | 2008 |
