# Supplementary material for: Vulnerability of Gubernatrix cristata to climate change, anthropogenic pressures, and hybridization threats
Source: Sci Rep. 2025 Apr 9;15:12152. doi: 10.1038/s41598-025-94293-7 (PMC11982183; doi:10.1038/s41598-025-94293-7)
Supplement: Supplementary file 8 — Supplementary Information 8. [file 41598_2025_94293_MOESM8_ESM.docx]

**Supplementary information**

**Assessing the vulnerability of the Yellow Cardinal (*Gubernatrix cristata*) to climate change, anthropogenic pressures, and hybridization threats**

Regina Gabriela Medina & Marisol Domínguez

**Supplementary text S1: Human Footprint index layer**

To investigate the level of anthropic pressure, we used the Human Footprint Index (HFP) from the NASA Data Center at a 1 km resolution scale [1]. This index combines built-up environments, human population density, electric power infrastructure, croplands, pasturelands, roads, railways, and navigable waterways [1]. In our study area, the HFP ranges from 0 (absolute natural environments) to 48 (high-density built environments). To obtain the areas with high values of HFP, we re-categorized data as low for values between 0 and 10, and high for values greater than 10.

[1] Venter, O. *et al.* Global terrestrial Human Footprint maps for 1993 and 2009. *Sci. Data* **3**, 160067. https://doi.org/10.1038/sdata.2016.67 (2016).

**Supplementary text S2: Protected areas layer**

To explore the current and future degree of protection of the yellow cardinal’s EGD, we quantified the proportion of its current and predicted range that falls within protected areas (PAs). We obtained this layer with PAs classified under IUCN management categories I to VI, ranging from strict nature reserves to areas allowing sustainable use of natural resources [2] from the *World Database on Protected Areas* (WDPA). Additionally, we included biosphere reserves and Ramsar sites due to their local importance for birds (Table S6). For Uruguay, we only considered PAs that belong to the National System of Protected Areas (SNAP). Finally, we gathered a shapefile with 63 current PAs.

[2] Dudley, N. *Guidelines for Applying Protected Area Management Categories*. (IUCN, Gland, Switzerland, 2008).

**Supplementary text S3: Estimations of geographic distributions and projections on future climate scenarios for *Diuca diuca***

Occurrence data for diucas, spanning 1972 to 2019, were obtained from the eBird database (https://ebird.org/home). We focused on records from August to February, covering the breeding season period when both species co-occur [3]. After removing duplicates, we spatially filtered records using a 30 km radius to reduce redundant localities, resulting in 493 records, which were divided 50% for testing and 50% for training the ecological niche model, using the R package *ellipsenm* [4]. For the abiotic component, we used bioclimatic variables from WorldClim (v1.4), selecting those that summarize temperature and precipitation information (correlation index r ≥ 0.85). The variables selected included average annual temperature, isothermality (bio2/bio7) (×100), temperature seasonality (standard deviation × 100), minimum temperature of the coldest month, annual precipitation, precipitation of the driest month, and precipitation seasonality (coefficient of variation). The area of calibration, model calibration, model selection, and future projections were performed as described for *G. cristata*. We set the threshold for generating binary presence-absence maps at 0.15. This represents the highest environmental suitability that predicts 85% of presence points used for calibration, accounting for a 15% dataset error.

[3] Narosky, T. & Yzurieta, D. *Guía Para La Identificación de Las Aves de Argentina y Uruguay.* (Asociación Ornitológica del Plata, Vázquez-Mazzini Editores, Buenos Aires, Argentina, 2003).

[4] Cobos, M. E. *et al.* ellipsenm: ecological niche’s characterizations using ellipsoids. R package version 0.3.4. at https://github.com/marlonecobos/ellipsenm (2022).

**Supplementary text S4: Inference of risk maps for the Yellow Cardinal**

To evaluate the potential risk to *Gubernatrix cristata*, we created two risk maps for 2050 and 2070, integrating predictions of both (1) geographic range loss, and (2) potential hybridization areas with *Diuca diuca* under future climate scenarios.

1. From each binary output for *G. cristata* (across each scenario and period), we quantified the number of sites (pixels) projected to be lost compared to the current EGD as follows:
2. We performed eight subtractions (present vs future for each of the four RCP scenarios for 2050 and 2070). The resulting output had three values: –1 (indicating a new site), 0 (absent) and 1 (a site expected to be lost).
3. Summing these results gave us a layer with values ranging from –4 to 4. We then reclassified this layer, converting negative values (indicating an increase in geographic rang size) to 0, and retaining positive values (1–4), where each number corresponds to the count of scenarios predicting geographic range loss.
4. To summarize the sites expected be lost in a single layer, we then sum these results, obtaining a layer whose values are –4 to 4. This was reclassified so that negative values (indicating an increase in geographic range size) turned into 0, and we only retained the positive values of 1 – 4, where 1 represents cells that will be lost in at least one scenario, 2 represents cells that will be lost in two scenarios, and so on up to 4.
5. We then summarize this through a consensus map in which each cell value quantified the number of times geographic range loss is predicted.
6. To assess the potential contact zone between *G. cristata* and *D. diuca***,**
7. we calculated the intersection of their EGDs in current and future climate scenarios (e.g., diuca_2650 ∩ cristata_2650 for scenario 2.6 for 2050).
8. we obtained a consensus map in which the values of each cell quantify the times when the contact zone is predicted.
9. We calculated the potential risk areas for each period throughout a weighted sum of the two layers (1) potential geographic range loss and (2) potential hybridization contact areas. We weighted geographic range loss with a factor of 0.7 and contact areas with 0.3.
10. Finally, we add a zero-risk term, calculated separately for the 2050 and 2070 periods, for areas that recover in all future scenarios and do not interact with *Diuca diuca*.

$$for 2050, 2070$$

$$\left( (geographic rangelost \times0.7+ potential contact hybridization zone \times0.3 \right)+zero-risk)\times10=$$

This approach allowed us to balance the importance of both factors in determining the overall risk of extinction. Geoprocessing and map edition were conducted using ArcGis Pro 3.2.2.
